# Using Multivalent Adenoviral Vectors for HIV Vaccination

**DOI:** 10.1371/journal.pone.0060347

**Published:** 2013-03-28

**Authors:** Linlin Gu, Zan C. Li, Alexandre Krendelchtchikov, Valentina Krendelchtchikova, Hongju Wu, Qiana L. Matthews

**Affiliations:** 1 Department of Medicine, Division of Infectious Diseases, University of Alabama at Birmingham, Birmingham, Alabama, United States of America; 2 Department of Medicine, Section of Endocrinology, Tulane University, New Orleans, Louisiana, United States of America; 3 Center for AIDS Research, University of Alabama at Birmingham, Birmingham, Alabama, United States of America; University of Pittsburgh, United States of America

## Abstract

Adenoviral vectors have been used for a variety of vaccine applications including cancer and infectious diseases. Traditionally, Ad-based vaccines are designed to express antigens through transgene expression of a given antigen. For effective vaccine development it is often necessary to express or present multiple antigens to the immune system to elicit an optimal vaccine as observed preclinically with mosaic/polyvalent HIV vaccines or malaria vaccines. Due to the wide flexibility of Ad vectors they are an ideal platform for expressing large amounts of antigen and/or polyvalent mosaic antigens. Ad vectors that display antigens on their capsid surface can elicit a robust humoral immune response, the “antigen capsid-incorporation” strategy. The adenoviral hexon protein has been utilized to display peptides in the majority of vaccine strategies involving capsid incorporation. Based on our abilities to manipulate hexon HVR2 and HVR5, we sought to manipulate HVR1 in the context of HIV antigen display for the first time ever. More importantly, peptide incorporation within HVR1 was utilized in combination with other HVRs, thus creating multivalent vectors. To date this is the first report where dual antigens are displayed within one Ad hexon particle. These vectors utilize HVR1 as an incorporation site for a seven amino acid region of the HIV glycoprotein 41, in combination with six Histidine incorporation within HVR2 or HVR5. Our study illustrates that these multivalent antigen vectors are viable and can present HIV antigen as well as His_6_ within one Ad virion particle. Furthermore, mouse immunizations with these vectors demonstrate that these vectors can elicit a HIV and His_6_ epitope-specific humoral immune response.

## Introduction

There has been a tremendous amount of progress with respect to infectious disease containment worldwide. However, safe and effective vaccines are needed to protect against many infections, including malaria, HIV, and tuberculosis. As it relates to recombinant adenovirus vaccine candidates against the pathogens mentioned, antigens are expressed as transgenes intracellularly after the vector infects a subset of cells. Alternatively, antigenic peptides can be delivered by recombinant vectors which present peptides on their capsid surface (fiber, pIX, and hexon). Ad vectors that display peptides on their surface can act as potent immunogens [1]–[10]. For effective vaccine development it is often necessary to express or present multiple antigens to the immune system to elicit an optimal vaccine as observed preclinically with mosaic/polyvalent HIV vaccines or malaria vaccines [5]–[7], [11]–[14]. Due to the wide flexibility of Ad vectors they are an ideal platform for expressing large amounts of antigen and/or polyvalent mosaic antigens [11], [15]. Routinely, these antigens are expressed as transgenes after cellular expression. Alternatively, these antigens can be displayed as exogenous peptides. Ad vectors that display antigens on their capsid surface can elicit a robust humoral immune response, this is known as the “antigen capsid-incorporation” strategy.

To increase the magnitude and/or breadth of antigen-specific antibody response, multiple capsid sites may be utilized. Adenovirus fiber [7], [16], penton base [16], pIX, [16]–[18] and hexon [2], [3], [7], [10], [19], [20] have been utilized for immune modulation by means of peptide incorporation. The adenoviral hexon protein has been utilized to display antigens in the majority of vaccine strategies involving capsid incorporation. The major capsid protein hexon has been utilized for these capsid incorporation strategies due to hexon's natural role in the generation of anti-Ad immune response and its numerical representation within the Ad virion (720 copies per virion).

As it relates to Ad serotype 2 hexon, hexon hypervariable region (HVR) 5 has been used to display antigens; in Ad serotype 5 (Ad5) hexon HVR1, HVR2, and HVR5 have been used to display antigens. To date, our group has been the only group to utilize Ad5 HVR2 for display of model [4] or disease-specific [5] antigens. Based on our abilities to manipulate HVR2 and HVR5, we sought to manipulate HVR1 in the context of HIV antigen display for the first time ever. More importantly, antigen incorporation within HVR1 was utilized in combination with antigen incorporation at other HVRs, thus creating multivalent vectors. Our definition of a multivalent vector is a vector that has the ability to vaccinate against several strains of an organism or vaccinate against two or more distinct organisms. In order to produce a multivalent vaccine vector, we generated vectors that display antigens within HVR1 and HVR2 or HVR1 and HVR5. Our study herein focuses on the generation of proof-of-concept vectors that can ultimately result in the development of multivalent vaccine vectors displaying dual antigens within the hexon of one Ad virion particle. To our knowledge this is the first successful demonstration achieving this goal. These novel vectors utilize HVR1 as an incorporation site for a seven amino acid epitope (ELDKWAS, which we will refer to as KWAS throughout this paper) of the HIV membrane proximal ectodomain region (MPER), derived from HIV glycoprotein 41 (gp41), in combination with a six Histidine (His_6_) incorporation within HVR2 or HVR5. Our report illustrates that our multivalent antigen vectors are viable and can present HIV antigen as well as His_6_ within one Ad virion particle. In addition, mouse immunizations with these vectors demonstrate that these vectors can elicit HIV and His_6_ epitope-specific humoral immune responses.

## Materials and Methods

### Antibodies

For these studies HIV-1 gp41 monoclonal antibody (2F5) was used. The following reagent was obtained through the NIH AIDS Research and Reference Reagent Program, Division of AIDS, NIAID, NIH: HIV-1 gp41 monoclonal antibody (2F5), cat# 1475 was generated by Dr. Hermann Katinger. The human monoclonal antibody to HIV-1 gp41 is specific for ELDKWA epitope [21]–[23]. This sequence is also genetically incorporated within the control vector Ad5/HVR2-MPER-L15ΔE1. Goat anti-human antibody conjugated to horseradish peroxidase (HRP) was purchased from Southern Biotech (Birmingham, AL). For the His_6_ studies, mouse anti-His_6_ monoclonal antibody was purchased from Qiagen (Valencia, CA). HRP-conjugated goat anti-mouse secondary antibody was purchased from DakoCytomation (Denmark). Isotype-specific goat anti-mouse antibodies were purchased from Sigma-Aldrich (St. Louis, MO). Donkey anti-goat HRP-conjugated antibody was purchased from Jackson ImmunoResearch Laboratories, Inc.

### Cell culture

Human embryonic kidney (HEK293) cells were obtained from and cultured in the medium recommended by the American Type Culture Collection (Manassas, VA). The cell line was incubated at 37°C and 5% CO_2_ under humidified conditions.

### Recombinant adenoviral construction

In order to generate recombinant adenoviruses with the KWAS epitope genetically incorporated within Ad hexon HVR1 as well as His_6_ incorporated within HVR2 or HVR5, the following was performed: for the HVR1 modification, the DNA sequence corresponding to a seven amino acid region of HIV gp41 was generated by GenScript and subcloned into the HVR1 region (the HIV sequence replaced amino acids 139 to 144) of the HVR2-His_6_/pH5S or HVR5-His_6_/pH5S plasmids [24]. In order to generate the control plasmid HVR1-His_6_/pH5S, the DNA sequence corresponding to His_6_ within the HVR1 (the His6 sequence replaced amino acids 139 to 144) was generated by GenScript and subcloned into the H5/pH5S plasmid [24]. The resulting plasmids HVR1-His_6_/pH5S, HVR1-KWAS-HVR2-His_6_/pH5S or HVR1-KWAS-HVR5-His_6_/pH5S were digested with EcoRI and PmeI. These resulting fragments containing the homologous recombination regions and the hexon genes were purified, then recombined with a SwaI-digested Ad5 backbone vector that lacks the hexon gene, pAd5/ΔH5 [25]. These recombination reactions were performed in Escherichia coli BJ5183 (Stratagene, La Jolla, CA). The resultant clones were designated as Ad/H5-HVR1-His_6_, Ad5/H5-HVR1-KWAS-HVR2-His_6_ and Ad5/H5-HVR1-KWAS-HVR5-His_6_. The control vector, Ad5/HVR2-MPER-L15ΔE1 was generated and characterized as previously described [5]. The Ad5 vector which is E1 deleted and contains green fluorescent protein and luciferase within the E1 region was generated and characterized as previously described [24].

### Vector rescue and preparation

To rescue vectors the constructed plasmids were digested with PacI and transfected with 3 µg DNA each (Lipofectamine 2000 Reagent, Invitrogen, Carlsbad, CA) into the Ad-E1-expressing HEK293 cells. Following plaque formation, they were processed for large-scale propagation in HEK293 cells. Vectors were purified by double cesium chloride ultracentrifugation and dialyzed against phosphate-buffered saline containing 10% glycerol. Vectors were stored at −80°C until use. Final aliquots of vectors were analyzed for physical titer using absorbance at 260 nm. The infectious particles (IP) per ml were determined by tissue culture infectious dose (TCID_50_) assay. The TCID_50_ titer was calculated by using KARBER statistical method: TCID_50_ titer  = 10×10^1+d(S−0.5)^/ml, in which d is the log 10 of the dilution and S is the sum of ratios from the first dilution. Modifications of the hexon gene was confirmed by PCR analysis with the primers 5′HVR2 (sense), CTCACGTATTTGGGCAGGCGCC and 3′HVR5 (antisense), GGCATGTAAGAAATATGAGTGTCTGGG, which anneal up and downstream of the site of the insertion within the hexon open reading frame.

### Western blot analysis

To analyze His_6_ display on selected vectors, 5×10^9^ viral particles (VPs) were boiled in Laemmli sample buffer for 10 minutes and resolved on 4 to 15% sodium dodecyl sulfate (SDS)-polyacrylamide gel. The proteins were transferred to polyvinylidene fluoride (PVDF) membrane and blotting was performed with penta-His monoclonal antibody (1∶2,000), followed by secondary incubation with HRP-conjugated goat anti-mouse antibody (1∶2,000). The proteins were detected on the PVDF membrane by using 3′3′-diaminobenzidine tablets (Sigma-Aldrich, St. Louis, MO) as substrate for HRP.

To analyze KWAS display on selected vectors, 5×10^9^ VPs were boiled in Laemmli sample buffer for 10 minutes and resolved on 4 to 15% SDS-polyacrylamide gel. The proteins were transferred to PVDF membrane and blotting was performed with HIV-1 gp41 monoclonal antibody (2F5) (1∶2,000), which was followed by secondary incubation with HRP-conjugated goat anti-human antibody (1∶2,000). The proteins were detected on the PVDF membrane by using 3′3′-diaminobenzidine tablets as substrate for HRP.

In brief, to analyze fiber expression within all Ad virions, 5×10^9^ VPs were boiled in Laemmli sample buffer for 10 minutes and resolved on 4 to 15% SDS-polyacrylamide gel. The proteins were transferred to PVDF membrane and blotting was performed with Anti-Adenovirus Fiber antibody [4D2] (1∶2000), followed by secondary incubation with HRP-conjugated goat anti-mouse antibody (1∶5,000). The proteins were detected on the PVDF membrane by using 3′3′-diaminobenzidine tablets as substrate for HRP.

### Mouse immunizations

The following experiments were performed to determine antibody response after immunization with Ad vectors. Female BALB/c (H-2Kd) mice at 6–8 weeks of age were obtained from the Charles River Laboratory. Groups of at least eight mice were immunized in each experiment or at each time point. Ads were injected into each group of mice at each time point by means of an intramuscular (i.m.) injection of 1×10^10^ VP. The interval between prime and boost was 21 days; the interval between boost and reboost was 18 days. The University of Alabama at Birmingham Institutional Animal Use and Care Committee approved the use of mice as described herein under the approved protocol number 101109272.

### Whole virus enzyme-linked immunosorbent assay (ELISAs) and serum ELISAs

For varied virus concentration-whole virus ELISAs, the ELISAs were performed essentially as described previously [25]. In order to determine if the His_6_ peptide was surface exposed on the Ad virion, whole virus ELISA was performed. Briefly, different amounts of vectors ranging from 2×10^6^ to 2.2×10^9^ VPs were immobilized on 96-well plates (Nunc Maxisorp, Rochester, NY) by overnight incubation in 100 µl of 100 mM carbonate buffer (pH 9.5) per well at 4°C. After washing with 0.05% Tween 20 in Phosphate-buffered saline (PBST) and blocking with blocking solution (5% bovine serum albumin in PBST), the immobilized vectors were incubated with anti-His_6_ monoclonal antibody (1∶2,000) for 2 hrs at room temperature (RT), followed by incubation with an HRP-conjugated goat anti-mouse antibody (1∶2,000). In order to determine if the KWAS peptide was surface exposed on the Ad virion, whole virus ELISA was performed in a similar fashion to the His_6_ ELISA. The immobilized viruses were incubated with HIV-1 gp41 monoclonal antibody (1∶2,000) for 2 hrs at RT followed by incubation with an HRP-conjugated goat anti-human antibody (1∶2,000).

For the varied antibody concentration-whole virus ELISAs, 6×10^8^ VP of vectors were immobilized on an ELISA plate followed by appropriate blocking, washing, and the addition of varying dilutions of either His_6_ antibody (1∶750; 1∶1,500; 1∶3,000; and 1∶6,000) or HIV antibody (1∶12,000; 1∶48,000; and 1∶192,000). The procedure was followed in a similar fashion as to the varied virus concentration-whole virus ELISAs. The binding was detected with the appropriate HRP-conjugated secondary antibodies as previously described.

For the serum-ELISAs (anti-His_6_ response), ELISA plates were coated with 1 µM of the His_6_ peptide (LGSHHHHHHLGS) (GenScript Co, Piscataway, NJ) in 100 µl of 50 mM carbonate (pH 9.5) per well as previously described in [5]. Plates were washed and then blocked with 5% BSA/PBST. After washing, 100 µl of diluted (1∶40; 1∶80; 1∶160; 1∶320; 1∶640; 1∶1,280; 1∶2,560) serum was added to the wells. After incubation for 2 hrs at RT, the plates were extensively washed and blocked again. Then the plates were incubated with goat anti-mouse secondary antibody (1∶2,000). The plates were washed again and the ELISAs were developed with SIGMAFAST OPD peroxidase substrate. OD 450 nm was measured on an Emax microplate reader.

For the serum-ELISAs (anti-HIV response), ELISA plates were coated with 1 µM of the KWAS (PCEWDEAELDKWASNLEEEDDDNE) peptide (GenScript Co, Piscataway, NJ) in 100 µl of 50 mM carbonate buffer (pH 9.5) per well as previously described in [5]. The experiment was performed in a similar fashion as the anti-His_6_ (serum-ELISAs) experiment, except goat anti-human secondary antibody (1∶2,000) was used.

In order to determine His_6_ or KWAS isotype-specific reactivity, ELISA plates were coated with 1 µM of the His_6_ or KWAS peptide in 100 µl of 50 mM carbonate buffer (pH 9.5) per well, according to the method we described previously in the above assays. Plates were washed and then blocked with 5% BSA/PBST. After washing, 100 µl of diluted (1∶40; 1∶80; 1∶160; 1∶320; 1∶640; 1∶1,280; 1∶2,560) serum was added to the wells. After incubation for 2 hrs at RT, the plates were extensively washed and blocked again. Various isotype-specific goat anti-mouse antibodies (1∶1,000) were then bound to ELISA plates. Plates were then washed and blocked, followed by the addition of donkey anti-goat HRP-conjugated antibody (1∶5,000) for 2 hrs at RT. The plates were washed and ELISAs were developed with SIGMAFAST OPD peroxidase substrate. OD 450 nm was measured on an Emax microplate reader.

### Statistical evaluation

The data were presented as the mean ± the standard deviation. Statistical analyses were performed with the nonpaired two-tailed Student t-test, assuming unequal variance. Statistical significance was defined as *P*≤0.05.

## Results

### Construction and characterization of multivalent Ad vectors

**Figure 1:**
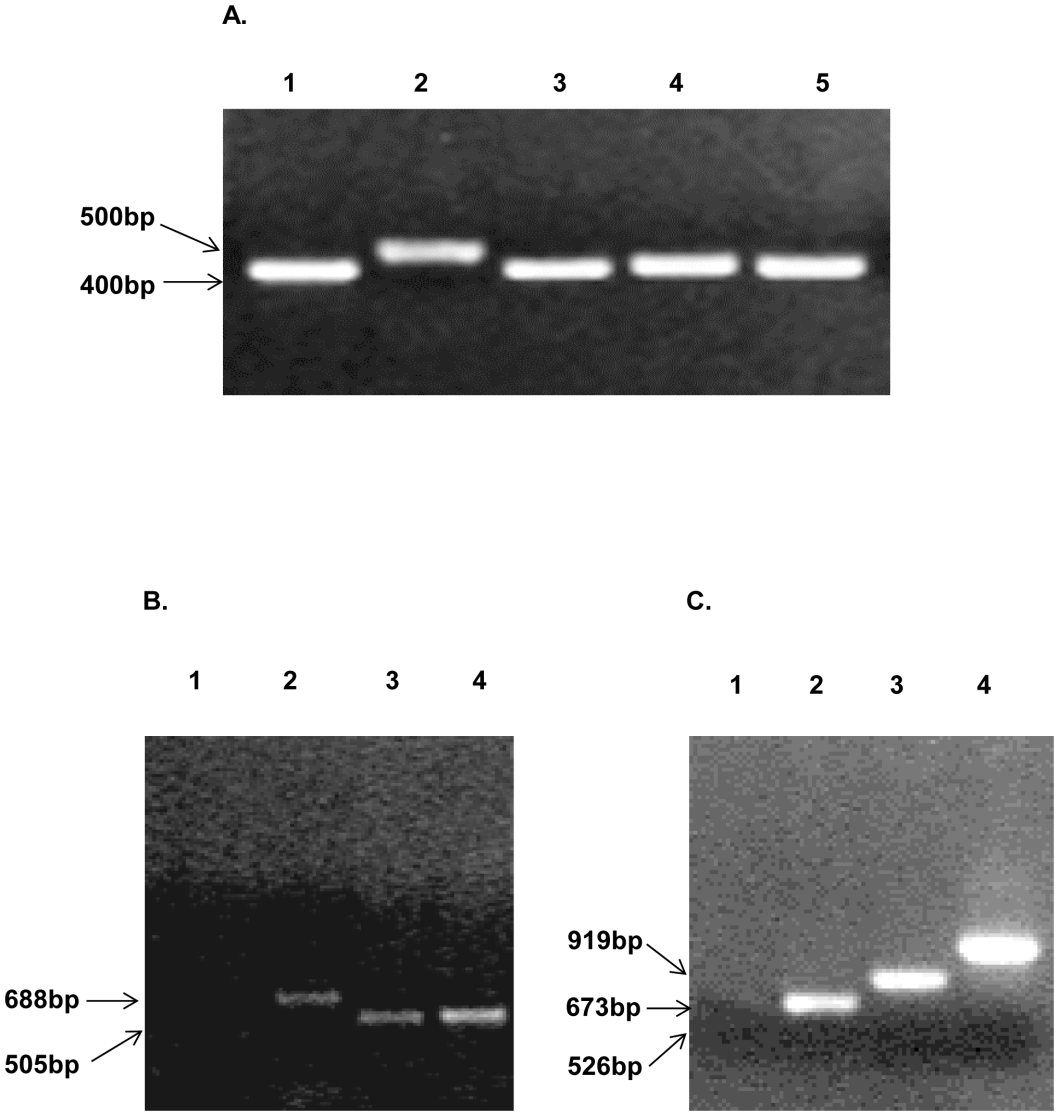
KWAS and His_6_ genetically incorporated into the HVR1 location as well as His_6_ within HVR2 or HVR5. Rescued vectors were amplified and viral DNA analyzed to confirm stable modification of relevant genes. A) Hexon-specific PCR primers confirmed the presence of the hexon gene in all of the modified vectors. Lane 1, Ad5; lane 2, Ad5/HVR2-MPER-L15ΔE1; lane 3, Ad/H5-HVR1-His_6_; lane 4, Ad5/H5-HVR1-KWAS-HVR2-His_6_; and lane 5, Ad5/H5-HVR1-KWAS-HVR5-His_6_. B) KWAS-specific primers confirmed the incorporation of coding regions for KWAS inserts in multivalent vectors. Lane 1, Ad5; lane 2, Ad5/HVR2-MPER-L15ΔE1; lane 3, Ad5/H5-HVR1-KWAS-HVR2-His_6_; and lane 4, Ad5/H5-HVR1-KWAS-HVR5-His_6_. C) His_6_-specific primers confirmed the incorporation of coding regions for His_6_ inserts in multivalent vectors. Lane 1, Ad5; lane 2, Ad/H5-HVR1-His_6_; lane 3, Ad5/H5-HVR1-KWAS-HVR2-His_6_; and lane 4, Ad5/H5-HVR1-KWAS-HVR5-His_6_. These vectors have been described in Table # 1.

After establishing the technical feasibilities allowing us to place model and disease-specific epitopes into HVR2 or HVR5 [4], [5] we sought to explore whether we could display dual antigens within Ad5 hexon in order to generate multivalent vaccine vectors. As an extension of our previous work [5] we choose to incorporate an HIV antigen (KWAS) within Ad hexon HVR1 as well as incorporate a model antigen, His_6_ within HVR2 or HVR5. The HIV antigen, KWAS was chosen as a disease-specific antigen to incorporate within the Ad hexon because it is derived from HIV envelope protein gp41. The gp41 envelope protein ectodomain is a target of three broadly neutralizing anti-HIV-1 antibodies [26]–[29]. His_6_ was chosen as a proof-of-concept antigen in order to determine if a double modification vector could be produced. For the HVR1 modifications, the DNA sequence corresponding to a seven amino acid epitope of HIV gp41 was subcloned into the HVR1 region (the HIV sequence replaced amino acids 139 to 144) of the HVR2-His_6_/pH5S or HVR5-His_6_/pH5S plasmids [24]. As indicated the His_6_ epitope was displayed at either the HVR2 or HVR5 position (Table #1). The resulting Ad genomes were partially sequenced to confirm that the correct genes were incorporated. Subsequent transfection of HEK293 cells with the recombinant genomes resulted in rescue of the following vectors: Ad/H5-HVR1-His_6_ (control vector), Ad5/H5-HVR1-KWAS-HVR2-His_6_ and Ad5/H5-HVR1-KWAS-HVR5-His_6_. In order to further confirm vector identities, hexon-specific PCR analyses were performed using genomic DNA from the purified virions (Figure 1A). Ad5 was found to have a wild type hexon PCR profile producing a 415 base pairs (bp) PCR fragment using the hexon-specific primers designed to amplify a region between HVR2 and HVR5 (Figure 1A, lane 1). Hexon-specific PCRs for controls Ad5/HVR2-MPER-L15ΔE1 and Ad/H5-HVR1-His_6_ gave a PCR profile of 534 and 414 bps (Figure 1A, lanes 2–3). Hexon-specific PCRs for Ad5/H5-HVR1-KWAS-HVR2-His_6_ and Ad5/H5-HVR1-KWAS-HVR5-His_6_ yielded PCR profiles of 438 and 423 bps (Figure 1A, lanes 4–5). The differences in bps among the vectors are related to deletions and insertions of DNA within the hexon genomes. KWAS-specific PCRs were used to confirm the presence of KWAS-specific DNA in the vector genomes. This was accomplished by utilizing a hexon-specific forward primer and a KWAS-specific reverse primer. This KWAS-specific PCR revealed a negative result for Ad5 (Figure 1B, lane 1). However, positive PCR results were seen for Ad5/HVR2-MPER-L15ΔE1, Ad5/H5-HVR1-KWAS-HVR2-His_6_ and Ad5/H5-HVR1-KWAS-HVR5-His_6_ (Figure 1B, lanes 2–4), suggesting the presence of KWAS-specific DNA within the Ad genomes. Next we sought to verify the incorporation of His_6_ sequences within Ad genomes. This was accomplished by utilizing a hexon-specific forward primer and a His_6_ reverse primer. His_6_-specific PCR on Ad5 virions revealed a negative result (Figure 1C, lane1). Whereas, hexon and His_6_-specific PCRs on Ad/H5-HVR1-His_6_, Ad5/H5-HVR1-KWAS-HVR2-His_6_ and Ad5/H5-HVR1-KWAS-HVR5-His_6_ viral DNA yielded positive results which varied from 526, 673, and 919 bps (Figure 1C, lanes 2–4). These results suggested that the His_6_ incorporations were present within the Ad genome; these variations were based on the placement of the His_6_ within the Ad HVRs. In summary, these data indicate that the incorporations of KWAS and His_6_ are present within the genomic DNA of Ad5/H5-HVR1-KWAS-HVR2-His_6_ and Ad5/H5-HVR1-KWAS-HVR5-His_6_. Having established the identities of the newly rescued Ad vectors, we next tested the impact of dual genetic incorporations on virus stability and/or infectious properties. Physical titers, as well as infectious titers were determined for each vector. The viral particle/infectious particle (VP/IP) ratio was calculated for all vectors. We observed a slightly increased VP/IP ratio for Ad/H5-HVR1-His_6_ as well as both dual antigen display vectors as compared to Ad5 (Table #2). These values are similar to what we observed in our previous 2008 study [4]. A normal VP/IP ratio of unmodified Ad ranges from ∼10–30.

**Table 1:** Description of vectors used in the study.

rAd Vectors	HVR1	HVR2	HVR5
Ad5	Unmodified	Unmodified	Unmodified
Ad5/HVR2-MPER-L15ΔE1	Unmodified	MPER	Unmodified
Ad/H5-HVR1-His6	His_6_	Unmodified	Unmodified
Ad5/H5-HVR1-KWAS-HVR2-His6	KWAS	His_6_	Unmodified
Ad5/H5-HVR1-KWAS-HVR5-His6	KWAS	Unmodified	His_6_

H5  =  hexon 5. All vectors are serotype and hexon 5-based

**Table 2:** Virological properties of vectors.

Modified Vectors	Viral Particles (VP)	Infectious Particles (IP)	VP/IP
Ad5	3.1×10^12^ vp/ml	1.0×10^11^ IP/ml	31
Ad5/HVR2-MPER-L15ΔE1	1.5×10^11^ vp/ml	1.3×10^8^ IP/ml	1,153
Ad/H5-HVR1-His6	2.0×10^12^ vp/ml	1.6×10^10^ IP/ml	125
Ad5/H5-HVR1-KWAS-HVR2-His6	2.0×10^12^ vp/ml	3.5×10^10^ IP/ml	57
Ad5/H5-HVR1-KWAS-HVR5-His6	1.3×10^11^ vp/ml	1.4×10^9^ IP/ml	92

### Display of dual antigens within Ad5 hexon hypervariable regions

**Figure 2:**
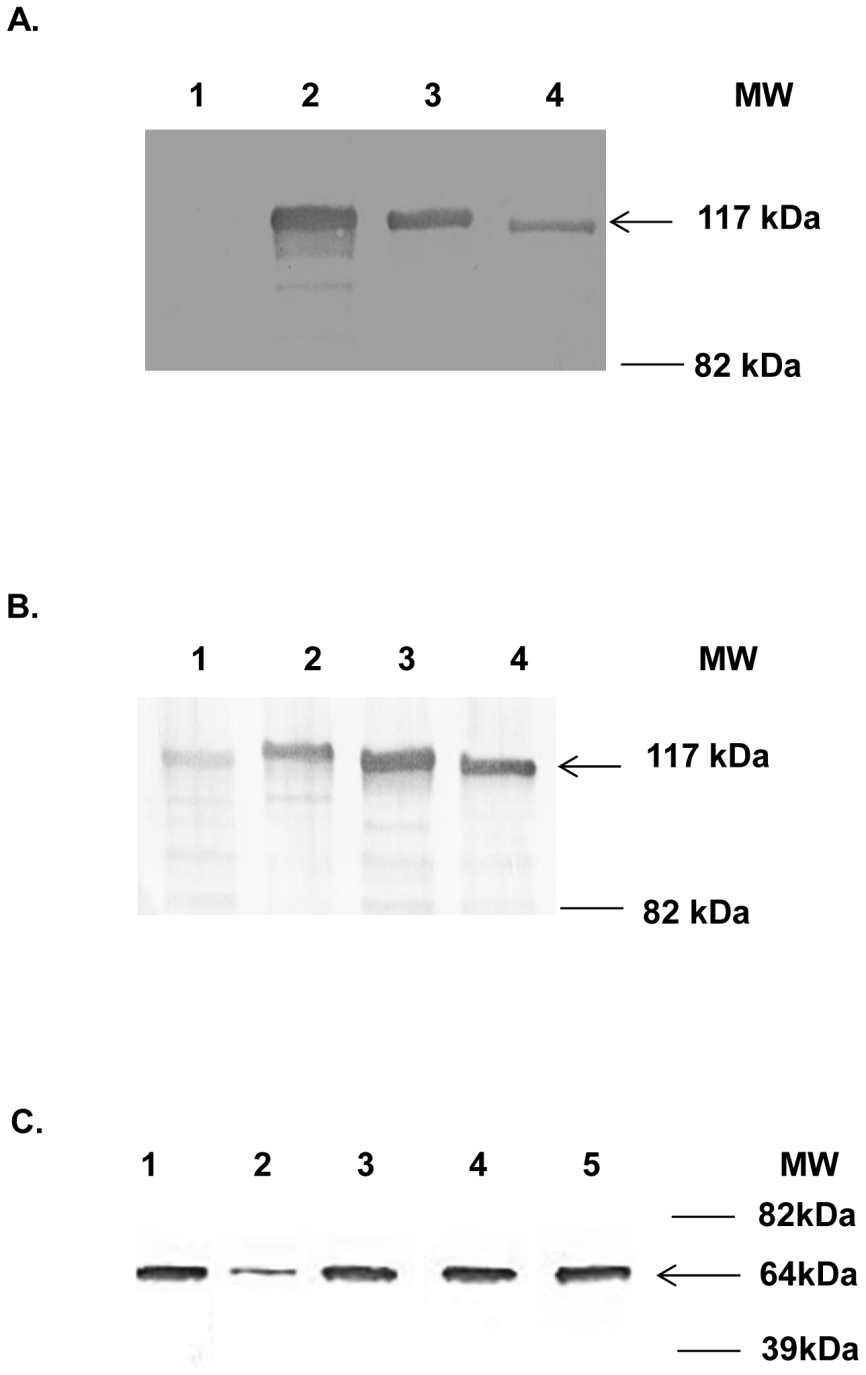
Western blotting confirmed the presence of His_6_, KWAS, and fiber on multivalent vaccine vectors. A) Western blotting confirmed the presence of His_6_ incorporation within the dual modified vectors. In this assay, 5×10^9^ VP of Ad5 (lane 1), Ad/H5-HVR1-His_6_ (lane 2), Ad5/H5-HVR1-KWAS-HVR2-His_6_ (lane 3), and Ad5/H5-HVR1-KWAS-HVR5-His_6_ (lane 4) were separated on 4 to 15% polyacrylamide gradient SDS-PAGE gel. The proteins were transferred to polyvinylidene fluoride (PVDF) membrane then incubated with anti-His antibody. The arrow indicates the His tag is genetically incorporated into the hexon protein. B) Western blotting confirmed the presence of KWAS incorporation within the dual modified vectors. In this assay, 5×10^9^ VP of Ad5 (lane 1), Ad5/HVR2-MPER-L15ΔE1 (lane 2), Ad5/H5-HVR1-KWAS-HVR2-His_6_ (lane 3), and Ad5/H5-HVR1-KWAS-HVR5-His_6_ (lane 4) were separated on 4 to 15% polyacrylamide gradient SDS-PAGE gel. The proteins were transferred to PVDF membrane then incubated with anti-KWAS antibody. The arrow indicates the KWAS epitope is genetically incorporated into the hexon protein. C) Western blotting confirmed the presence of fiber incorporation within the dual modified vectors. In this assay, 5×10^9^ VP of Ad5 (lane 1), Ad5/HVR2-MPER-L15ΔE1 (lane 2), Ad/H5-HVR1-His_6_ (lane 3), Ad5/H5-HVR1-KWAS-HVR2-His_6_ (lane 4), and Ad5/H5-HVR1-KWAS-HVR5-His_6_ (lane 5) were separated on 4 to 15% polyacrylamide gradient SDS-PAGE gel. The proteins were transferred to PVDF membrane then incubated with anti-Fiber antibody. The arrow indicates detection of the fiber protein.

After successful incorporation of dual antigen genes, we next sought to verify expression of our incorporations at the protein level by Western blot analysis. In order to determine if the hexon-modified vectors were displaying dual antigens within the hexon region, purified unmodified Ad5 (control vector), Ad5/HVR2-MPER-L15ΔE1 (control vector), Ad/H5-HVR1-His_6_ (control vector), Ad5/H5-HVR1-KWAS-HVR2-His_6_ and Ad5/H5-HVR1-KWAS-HVR5-His_6_ were subjected to Western blot analysis with anti-His_6_ and anti- MPER/KWAS antibodies. The His_6_ protein was detected as a 117 kDa protein band associated with Ad/H5-HVR1-His_6_ Ad5/H5-HVR1-KWAS-HVR2-His_6_, and Ad5/H5-HVR1-KWAS-HVR5-His_6_ particles (Figure 2A, lanes 2, 3, and 4, respectively). The size of the 117 kDa band corresponds to the expected size of the Ad5 hexon protein with His_6_ peptide genetically incorporated into the HVR1, HVR2, or HVR5 locations (control vector, Ad/H5-HVR1-His_6_). There was no His_6_ protein detected on Ad5 wild type particles (Figure 2A, lane 1).

The MPER/KWAS protein was detected as a 117 kDa protein band associated with Ad5/HVR2-MPER-L15ΔE1, Ad5/H5-HVR1-KWAS-HVR2-His_6_, and Ad5/H5-HVR1-KWAS-HVR5-His_6_ particles (Figure 2-B, lanes 2, 3, and 4, respectively). The size of the 117 kDa band corresponds to the expected size of the Ad5 hexon protein with MPER/KWAS peptide genetically incorporated into the HVR1 or HVR2 (Ad5/HVR2-MPER-L15ΔE, control vector) locations. There was a slight protein response detected on Ad5 wild type particles (Figure 2B, lane 1). Most importantly, these data indicate (Figure 2A and B) that KWAS and His_6_ were incorporated at comparable levels within HVR1 and HVR2 or HVR5.

As a control experiment, we evaluated whether multivalent vectors were expressing another capsid protein as normal or less than unmodified vectors. In brief, purified unmodified Ad5 (control vector), Ad5/HVR2-MPER-L15ΔE1 (control vector), Ad/H5-HVR1-His_6_ (control vector), Ad5/H5-HVR1-KWAS-HVR2-His_6_ and Ad5/H5-HVR1-KWAS-HVR5-His_6_ were subjected to Western blot analysis with anti-Fiber antibody. The fiber protein was detected as a monomer at a 64 kDa protein band associated with all the vectors. Notably, the relative fiber expression levels for the double modified vectors are comparable to that of the control vectors (Figure 2C).

### Dual antigens incorporated within HVRs are exposed on the virion surface

**Figure 3:**
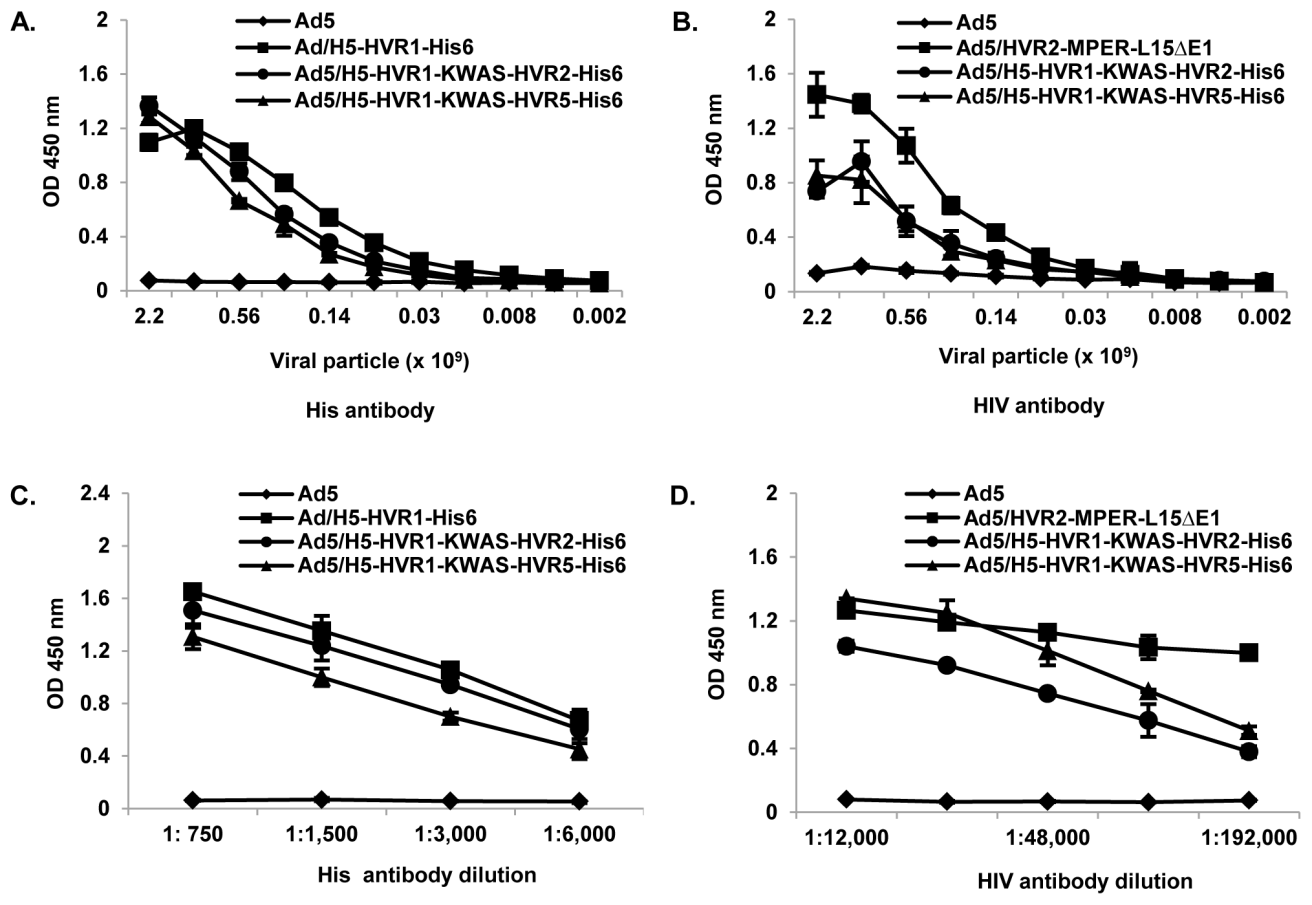
HIV and His_6_ epitopes in the HVRs are exposed on the virion surface. A) In the assay, varying amounts of Ad5, Ad/H5-HVR1-His_6_, Ad5/H5-HVR1-KWAS-HVR2-His_6_, or Ad5/H5-HVR1-KWAS-HVR5-His_6_ were immobilized in the wells of ELISA plates and incubated with anti-His_6_ antibody. The binding was detected with an HRP-conjugated secondary antibody. B) In the assay, varying amounts of Ad5, Ad5/HVR2-MPER-L15ΔE1, Ad5/H5-HVR1-KWAS-HVR2-His_6_, or Ad5/H5-HVR1-KWAS-HVR5-His_6_ were immobilized in the wells of ELISA plates and incubated with anti-HIV antibody. The binding was detected with an HRP-conjugated secondary antibody. C) In the assay 6×10^8^ VP of Ad5, Ad/H5-HVR1-His_6_, Ad5/H5-HVR1-KWAS-HVR2-His_6_, or Ad5/H5-HVR1-KWAS-HVR5-His_6_ were immobilized on an ELISA plate followed by varying dilutions of His_6_ antibody. The binding was detected with an HRP-conjugated secondary antibody. D) In the assay 6 x 10^8^ VP of Ad5, Ad5/HVR2-MPER-L15ΔE1, Ad5/H5-HVR1-KWAS-HVR2-His_6_, or Ad5/H5-HVR1-KWAS-HVR5-His_6_ were immobilized on an ELISA plate followed by varying dilutions of HIV antibody. The binding was detected with an HRP-conjugated secondary antibody.

These studies validated our ability to derive stable vectors that incorporate KWAS and His_6_ antigens within one virion particle. To this end, we performed whole virus ELISA assays to verify that the KWAS and His_6_ motifs were accessible on the virion surface. In this assay, varying amounts of purified vectors were immobilized in the wells of an ELISA plate and incubated with anti-His_6_ antibody. The results showed significant binding of the anti-His_6_ antibody to the Ad/H5-HVR1-His_6_ (control vector), Ad5/H5-HVR1-KWAS-HVR2-His_6_ and Ad5/H5-HVR1-KWAS-HVR5-His_6_, whereas no binding was seen in response to Ad5 control. These results indicate that the His_6_ epitope was properly exposed on the virion surfaces when incorporated within HVR2 or HVR5 (Figure 3A). We performed an ELISA assay to verify that the HIV motif was accessible on the virion surface within the HVR1 region (Figure 3B). In this assay, varying amounts of purified vectors were immobilized in the wells of an ELISA plate and incubated with anti-MPER/KWAS antibody. The results showed significant binding of the anti-HIV antibody to the Ad5/HVR2-MPER-L15ΔE1 (control vector), Ad5/H5-HVR1-KWAS-HVR2-His_6_ and Ad5/H5-HVR1-KWAS-HVR5-His_6_, whereas no binding was seen in response to Ad5 control.

In order to determine the capability of the His_6_ or HIV-specific antibodies to bind capsid-incorporated antigen in a dose-dependent manner, dose-response ELISA assays were performed with anti-His_6_ or anti-HIV antibodies. The following vectors: Ad5, Ad/H5-HVR1-His_6_, Ad5/H5-HVR1-KWAS-HVR2-His_6_ and Ad5/H5-HVR1-KWAS-HVR5-His_6_ were immobilized in a single concentration on ELISA plates, followed by the addition of serial dilutions of anti-His_6_ antibody. As predicted, the anti-His_6_ antibody bound to Ad/H5-HVR1-His_6_, Ad5/H5-HVR1-KWAS-HVR2-His_6_ and Ad5/H5-HVR1-KWAS-HVR5-His_6_ in a dose-dependent manner (Figure 3C). Our data suggest that the His_6_ epitope is presented within the hexon at HVR2 or HVR5 regions. Next we sought to determine if HIV-specific antibody bound capsid-incorporated antigen in a dose-dependent manner. The following vectors: Ad5, Ad5/HVR2-MPER-L15ΔE1, Ad5/H5-HVR1-KWAS-HVR2-His_6_ and Ad5/H5-HVR1-KWAS-HVR5-His_6_ were immobilized in single concentration on ELISA plates, followed by the addition of serial dilutions of anti-HIV antibody. As expected, the anti-HIV antibody bound to Ad5/HVR2-MPER-L15ΔE1, Ad5/H5-HVR1-KWAS-HVR2-His_6_ and Ad5/H5-HVR1-KWAS-HVR5-His_6_ in a dose-dependent manner (Figure 3D). Our data indicate that the KWAS epitope is presented within the hexon in its native conformation as it can be recognized by a monoclonal HIV neutralizing antibody.

### Anti-His_6_ antibodies produced in mice after immunization with multivalent vectors

**Figure 4:**
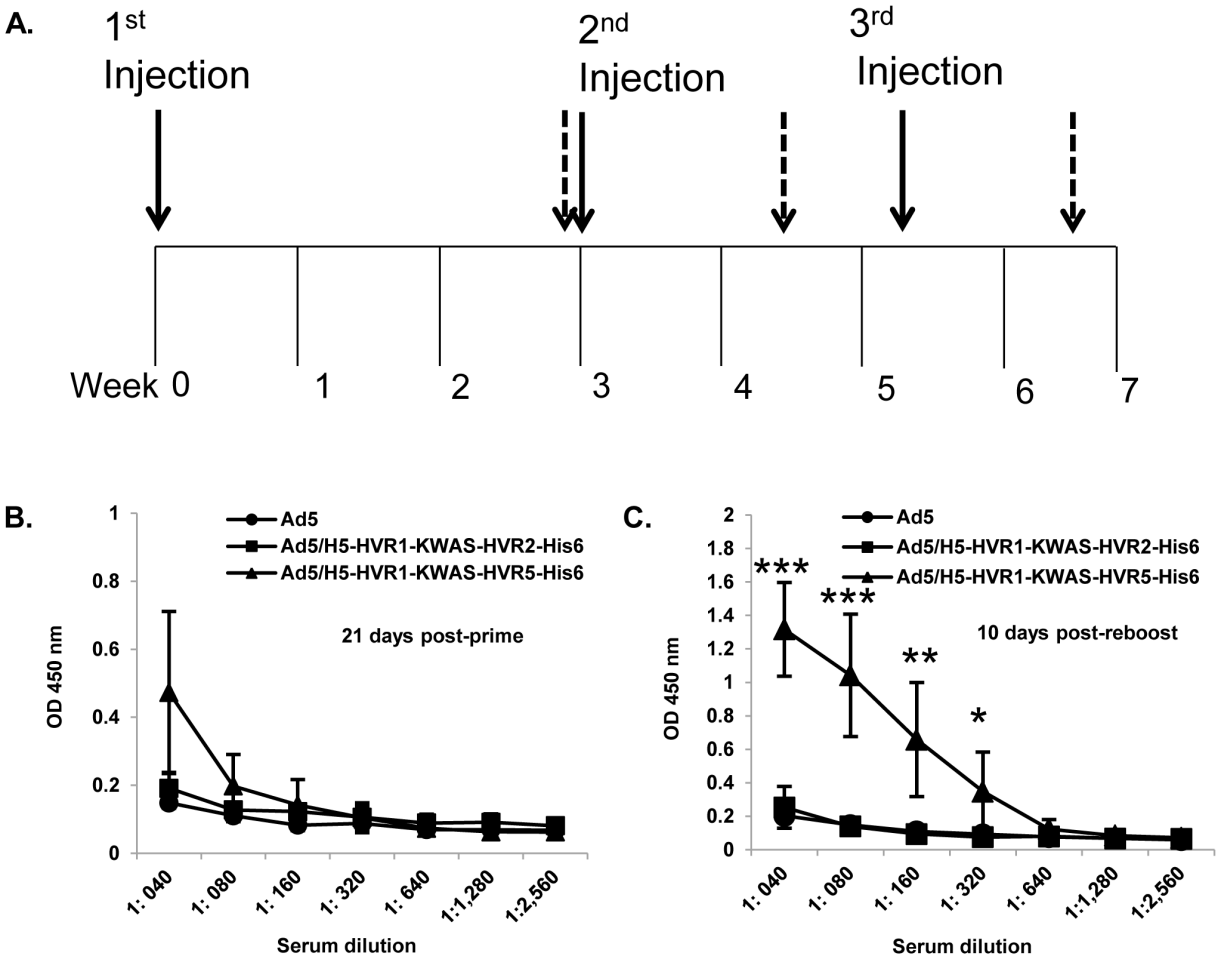
Multivalent vectors elicit an *in vivo* anti-His_6_ immune response. BALB/c mice (n = 8) were primed, boosted, and reboosted with 1×10^10^ VP of Ad vectors. A) Immunization timeline showing when immunizations were performed (solid arrows); serum was collected (dashed arrows). B–C) Post-prime and post-reboost serum was collected for ELISA binding assays. 1 µM of purified His_6_ (LGSHHHHHHLGS) antigenic peptide was bound to ELISA plates. Residual unbound peptide was washed from the plates. The plates were then incubated with varying concentrations of immunized mice serum and the binding was detected with HRP conjugated secondary antibody. OD absorbance at 450 nm represents His_6_ antibody levels in serum. The values are expressed as the mean ± standard deviation. The asterisks (***) indicate a *P* value ≤ 0.001, ** indicate a *P* value ≤ 0.01, and * indicate a *P* value ≤ 0.05.

We next sought to determine if our multivalent vectors were capable of eliciting an anti-His_6_ response in mice (Figure 4A). 1×10^10^VP were used to immunize BALB/c mice via an intramuscular (i.m.) route. The serum was collected from mice at 21 days post-prime, 13 days post-boost, and 10 days post-reboost. Purified His_6_ antigenic peptide was bound to ELISA plates. The plates were then incubated with varying concentrations of immunized mice sera. The binding was detected with HRP-conjugated secondary antibody. The data demonstrate no binding of the His_6_ antigenic peptide to the sera from mice immunized with Ad5 or Ad5/H5-HVR1-KWAS-HVR2-His_6_ at any time point or sera concentration (4B and C). In contrast, immunization with Ad5/H5-HVR1-KWAS-HVR5-His_6_ elicits an anti-His_6_-specific response. This finding was evident at 13 days post-boost (data not shown) and 10 days post-reboost, the His_6_-specific immune response seen after immunization with Ad5/H5-HVR1-KWAS-HVR5-His_6_ was significant in comparison to Ad5 immunization at 13 days post-boost (data not shown). The His_6_-specific immune response seen after immunization with Ad5/H5-HVR1-KWAS-HVR5-His_6_ was significant in comparison to Ad5 immunization at 10 days post-reboost (significance at the following serum dilutions: 1∶40, *p* ≤ 0.001; 1∶80, *p* ≤ 0.001; 1∶160, *p* ≤ 0.01; 1∶320, *p* ≤ 0.05) (Figure 4C).

### Anti-KWAS antibodies produced in mice after immunization with multivalent vectors

**Figure 5:**
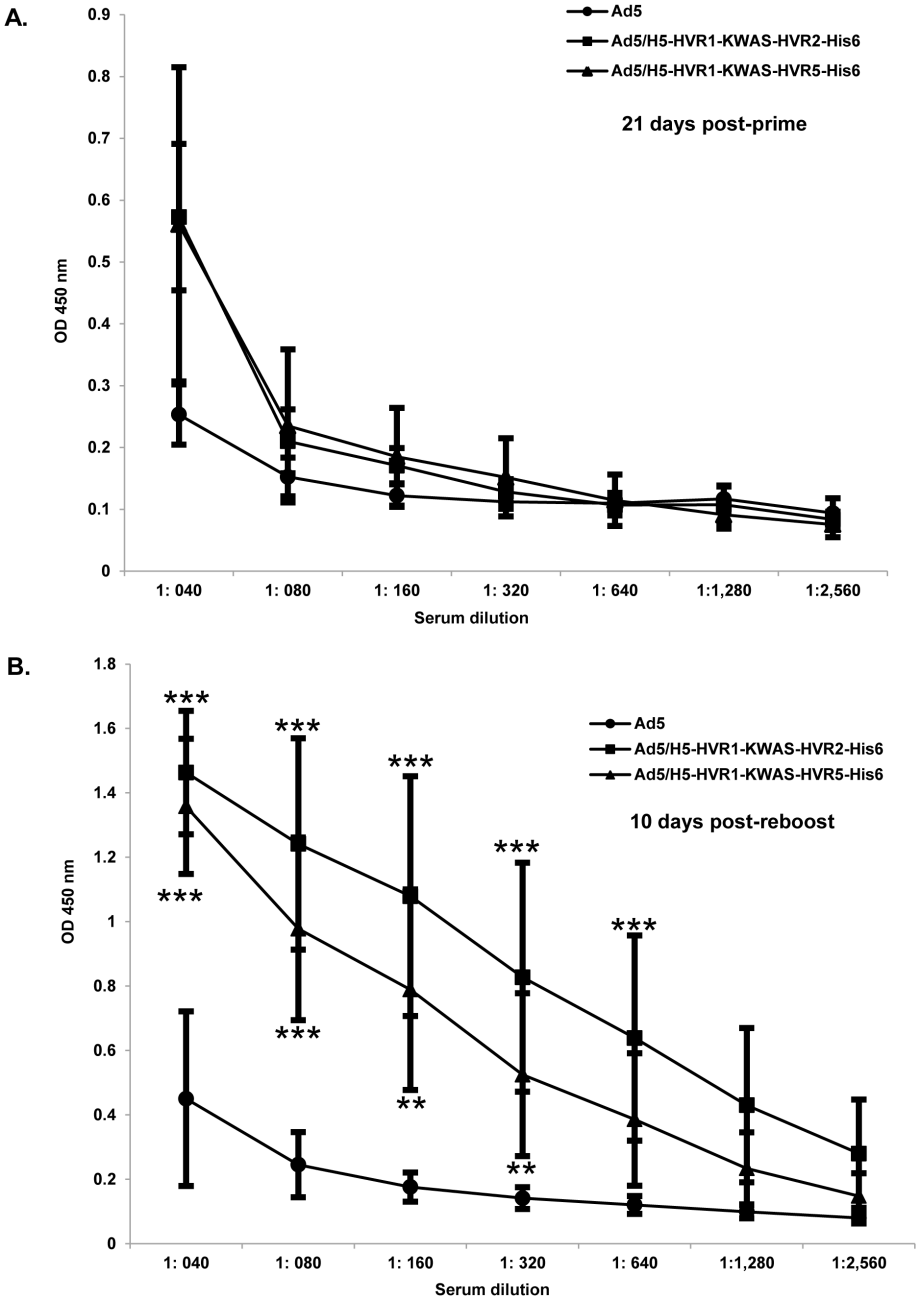
Multivalent vectors elicit an *in vivo* anti-KWAS immune response. BALB/c mice (n = 8) were primed, boosted, and reboosted with 1×10^10^ VP of Ad vectors (Figure 4A). A–B) Post-prime and post-reboost serum was collected at various time points for ELISA binding assays. 1 µM of purified KWAS (PCEWDEAELDKWASNLEEEDDDNE) antigenic peptide was bound to ELISA plates. Residual unbound peptide was washed from the plates. The plates were then incubated with immunized mice serum and the binding was detected with HRP conjugated secondary antibody. OD absorbance at 450 nm represents KWAS antibody levels in sera. The values are expressed as the mean ± standard deviation. The *** indicate a *P* value ≤ 0.001, and ** indicate a *P* value ≤ 0.01.

We next sought to determine if our multivalent vectors were capable of eliciting an anti-KWAS response in mice (Figure 4A). In these experiments purified KWAS antigenic peptide was bound to ELISA plates. The plates were then incubated with the immunized mice serum. The binding was detected with HRP-conjugated secondary antibody. The data demonstrate no binding of the KWAS peptide to the serum from mice immunized with Ad5 at any time point or serum concentration. Immunization with Ad5/H5-HVR1-KWAS-HVR2-His_6_ or Ad5/H5-HVR1-KWAS-HVR5-His_6_ yielded only a slight increase of binding to the KWAS peptide with serum from 21 days post-prime in comparison to Ad5 (Figure 5A). In contrast, Ad5/H5-HVR1-KWAS-HVR2-His_6_ or Ad5/H5-HVR1-KWAS-HVR5-His_6_ yielded substantial anti-KWAS-specific responses at 13 days post-boost (data not shown) and 10 days post-reboost (Figure 5B). Ad5/H5-HVR1-KWAS-HVR2-His_6_ and Ad5/H5-HVR1-KWAS-HVR5-His_6_ responses were significant in comparison to Ad5 immunization at 13 days post-boost (data not shown). Ad5/H5-HVR1-KWAS-HVR2-His_6_ and Ad5/H5-HVR1-KWAS-HVR5-His_6_ responses were significant in comparison to Ad5 immunization at 10 days post- reboost (Figure 5B).

### Isotype-specific anti-His_6_ and anti-KWAS antibodies are produced in mice after immunization

**Figure 6:**
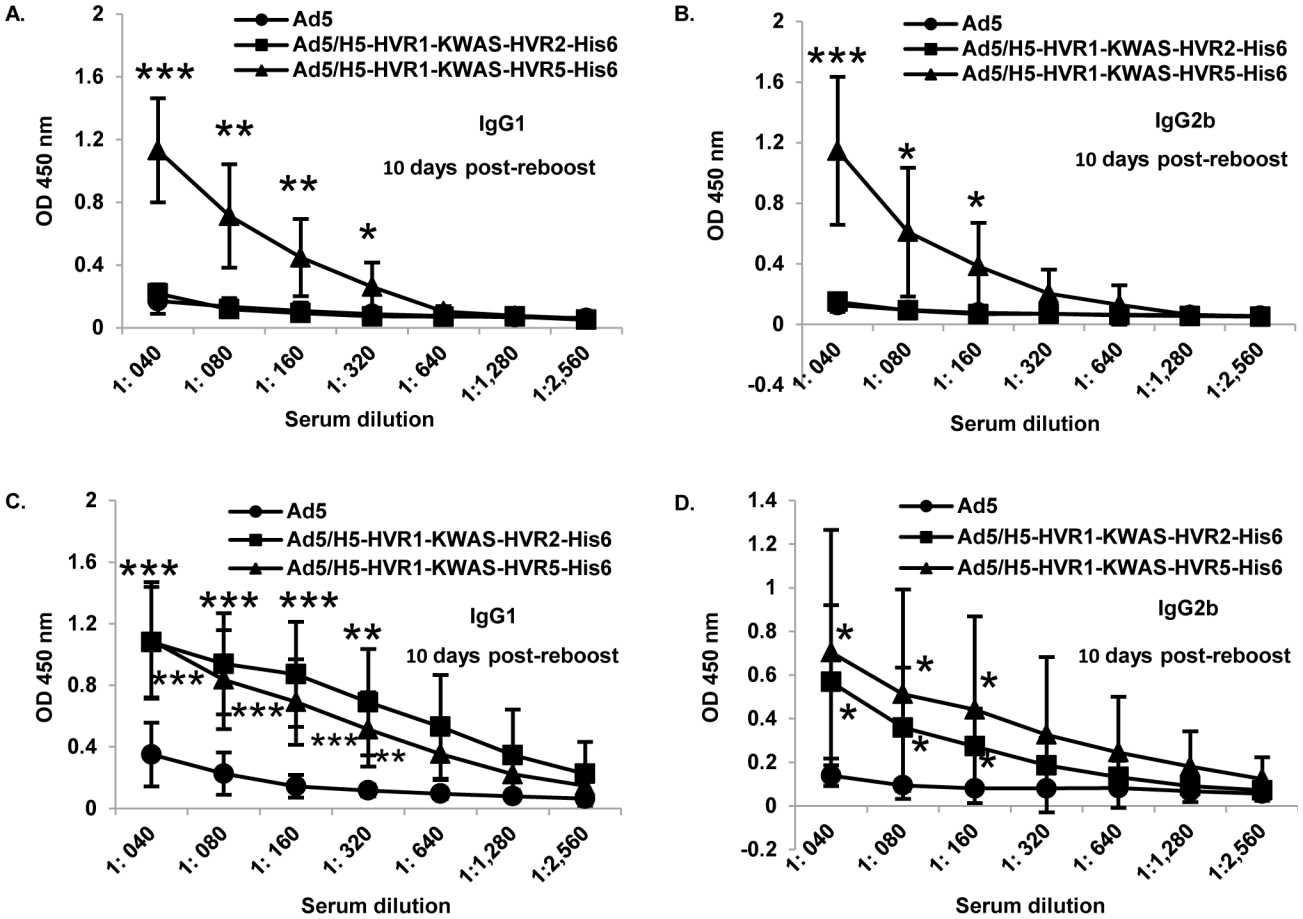
Multivalent vectors elicit *in vivo* His_6_ and KWAS isotype-specific responses. BALB/c mice (n = 8) were primed, boosted, and reboosted with 1×10^10^ VP of Ad vectors (Figure 4A). 10 days post-reboost serum was used for the isotype-specific assays. A–B) 1 µM of purified His_6_ (LGSHHHHHHLGS) antigenic peptide or C–D) 1 µM of purified KWAS (PCEWDEAELDKWASNLEEEDDDNE) antigenic peptide was bound to ELISA plates. Residual unbound peptide was washed from the plates. The plates were then incubated with immunized mice serum followed by isotype-specific antibodies. The binding was detected with HRP conjugated secondary. OD at 450 nm represents isotype-specific His_6_ or KWAS antibody levels in sera. The values are expressed as the mean ± standard deviation. The *** indicate a *P* value ≤ 0.001, ** indicate a *P* value ≤ 0.01, and * indicate a *P* value ≤ 0.05.

We next sought to evaluate the isotype-specific humoral immune responses after vector immunizations, anti-His_6_ (Figure 6A and B) and anti-HIV (Figure 6C and D). We analyzed sera from our previous assays at 10 days post-reboost. Purified His_6_ or KWAS peptides were bound to ELISA plates, respectively. The plates were then incubated with immunized mouse serum, followed by isotype-specific antibodies, IgG1 and IgG2b, respectively. The data illustrate anti-His_6_ isotype-specific responses at the reboost time point in animals immunized with Ad5/H5-HVR1-KWAS-HVR5-His_6_ (Figure 6A and B), similar to the trend seen in Figure 4. Ad5/H5-HVR1-KWAS-HVR5-His_6_ mediated anti-His_6_ isotype-specific responses were significant in comparison to Ad5 immunization at 10 days post-reboost. In contrast, there was no anti-His_6_ isotype-specific responses seen in animals immunized with either Ad5 or Ad5/H5-HVR1-KWAS-HVR2-His_6_, also similar to the trend seen in Figure 4. The data illustrate anti-KWAS isotype-specific responses at the reboost time point in animals immunized with Ad5/H5-HVR1-KWAS-HVR2-His_6_ and Ad5/H5-HVR1-KWAS-HVR5-His_6_ (Figure 6C and D) similar to the trend seen in Figure 5B. Both multivalent vectors mediated anti-KWAS isotype-specific responses which were significant in comparison to Ad5 immunization at 10 days post-reboost. There was no anti-KWAS isotype-specific responses observed in animals immunized with Ad5 vector.

## Discussion

We have developed novel Ad vectors that have the potential to optimize Ad vaccine approaches. This strategy involves inserting multiple antigenic epitopes within various hexon HVRs. Our current data demonstrate for the first time ever that multiple antigens can be incorporated in combination at two sites within the major capsid protein, hexon. Based on our abilities to manipulate HVR2 and HVR5, we sought to manipulate HVR1 in the context of HIV antigen display for the first time ever. More importantly, antigen incorporation within HVR1 was utilized in combination with antigen incorporation at other HVRs. In order to create a multivalent vaccine vector, we created vectors that display antigens within HVR1 and HVR2 or HVR1 and HVR5. In this study we successfully incorporated an HIV epitope within Ad5 HVR1, in combination with an incorporation of His_6_ epitope within Ad5 HVR2 or Ad5 HVR5 as proven by PCR, Western analysis, and whole-virus ELISAs (Figures 1, 2, and 3). Of note, these vectors have normal virological properties (Table #2). Most importantly, our *in vivo* studies illustrate that we achieved epitope-specific responses against our vectors after immunization (Figures 4, 5, and 6).

*In vitro* analysis of these multivalent vectors appears relatively comparable. For instance, His_6_ and KWAS incorporations as well as fiber protein appear to be relatively equal for Ad5/H5-HVR1-KWAS-HVR2-His_6_ and Ad5/H5-HVR1-KWAS-HVR5-His_6_ vectors (Figure 2). Along these same lines, whole virus ELISAs yield similar results for Ad5/H5-HVR1-KWAS-HVR2-His_6_ and Ad5/H5-HVR1-KWAS-HVR5-His_6_ when varying amounts of virus or antibody is compared (Figure 3). In contrast, we observed immunological differences associated with *in vivo* immune responses against Ad5/H5-HVR1-KWAS-HVR2-His_6_ or Ad5/H5-HVR1-KWAS-HVR5-His_6_ vectors. In this regard, after immunization we observed that the Ad5/H5-HVR1-KWAS-HVR5-His_6_ vector was able to elicit a His_6_ and KWAS antigen-specific total immunoglobulin response as well as His_6_ and KWAS isotype-specific immune responses (Figures 4, 5, and 6); whereas immunization with Ad5/H5-HVR1-KWAS-HVR2-His_6_ vector, yielded only KWAS antigen-specific total immunoglobulin response and KWAS isotype-specific immune response (Figures 4, 5, and 6).

In an independent set of experiments we incorporated dual antigens (Flag and His_6_) within HVR2 and HVR5 on a single Ad virion, Ad5/H5-HVR2-Flag-HVR5-His_6_. We also designed a second vector where the antigens were in the reverse configuration (His_6_ and Flag) within HVR2 and HVR5, Ad5/H5-HVR2-His_6_-HVR5-Flag. Both vectors rescued at a relatively normal growth rate; however, when analyzing these vectors by Western blot analysis we observed detrimental effects that could only be attributed to the dual antigen modifications. Western blot analysis of Flag and His_6_ incorporated within Ad5/H5-HVR2-Flag-HVR5-His_6_, yielded protein detection of Flag; however, we were unable to detect His_6_ associated with these particles (data not shown). Western blot analysis of His_6_ and Flag incorporated within Ad5/H5-HVR2-His_6_-HVR5-Flag, yielded protein detection of His_6_ and Flag. However, the protein expression of both antigens were not as equal as observed with our multivalent vectors (data not shown). In order to determine the minimal threshold of antigen required for immune activation, we speculate that it would still be beneficial to perform *in vivo* immunization with Ad5/H5-HVR2-His_6_-HVR5-Flag to evaluate epitope-specific immune response as well as cryoEM or molecular modeling with this vector.

Our multivalent vector study is a logical extension of our 2008 study. In our 2008 study we evaluated the use of Ad5 HVR2 or HVR5 vectors containing identical antigenic epitopes in either region. To compare the capacities and flexibility of Ad5 HVR2 to those of HVR5, we genetically incorporated identical epitopes of increasing size within HVR2 or HVR5 of the Ad5 hexon. The epitopes ranged in size from 33–83 amino acids. Stable vectors were produced with incorporations of 33 amino acids plus a 12 amino acid linker at HVR2 or HVR5. In addition, stable vectors were produced with incorporations of up to 53 amino acids plus a 12 amino acid linker in HVR5. HVR5 was more permissive, allowing an epitope incorporation of 65 amino acids. Whole virus ELISA analysis revealed that the model antigens were virion surface-exposed, and *in vivo* immunization with these vectors elicited antigen-specific immune responses.

Our findings herein are timely because it is the first time to our knowledge that multiple antigens have been incorporated on a single virion in combination at two sites within the major capsid protein, hexon. Substantial differences were observed when identical antigens were incorporated within the hexon protein. This finding is important for rationale vector design. Immunization with Ad5/H5-HVR1-KWAS-HVR2-His_6_, could not yield a dual antigen response against both antigens incorporated within HVR1 and HVR2. Only an antigen-specific immune response could be observed against antigen incorporated within HVR1. These results are contrary to previous studies which illustrated that a robust immune response could be elicited against His_6_ incorporated within HVR2 [4], [24]. Therefore, the combination of antigens incorporated within HVR1 and HVR2 must yield detrimental effects for *in vivo* immune presentation of the antigen at the HVR2 locale. However, immunizations with the Ad5/H5-HVR1-KWAS-HVR5-His_6_ vector yielded dual immune response against both antigens incorporated within HVR1 and HVR5. This finding is important because it illustrates that one Ad vector can be used to vaccinate against two potential antigens, achieving nearly equivalent immunizations.

Preclinically, incorporating antigens into viral capsid structures has been used as a vaccination approach for several diseases [1]-[4], [8], [16]. In 1994, Crompton and colleagues used this strategy for the first time in the context of Ad [1]. Crompton's group genetically incorporated an 8 amino acid sequence of the VP1 capsid protein of poliovirus type 3 into 2 regions of the adenovirus serotype 2 hexon. One vector produced from this attempt was able to grow well in tissue culture, and antiserum raised against the Ad with the polio antigen specifically recognized the VP1 capsid of the polio virus. Of note in 1999, an epitope was incorporated within Ad5 HVR5 for the purpose of tropism modifications. Vigne and colleagues incorporated an RGD motif within Ad5 HVR5 in order to investigate whether hexon-modified capsids would enhance the transduction of cells displaying limiting amounts of the virus attachment receptors. Interestingly, although hexon has never been implicated in Ad entry, the modified virus significantly increased the transduction of human vascular smooth muscle cells *in vitro* [30]. Worgall and colleagues used the antigen capsid-incorporation strategy to vaccinate against Pseudomonas aeruginosa (*pseudomonas*), a Gram-negative bacteria that causes respiratory tract infections in individuals who are immunocompromised or who have cystic fibrosis [31]. Because *pseudomonas* is an extracellular pathogen, anti-*pseudomonas* humoral immunity should be sufficient to provide protective immunity. Therefore, *pseudomonas* can be a candidate agent for vaccine development. Several immunogenic peptides have been identified in the outer membrane protein F (OprF) of *pseudomonas*, including the immunodominant 14-mer peptide Epi8. This study characterized genetic incorporations of a neutralizing epitope from the *pseudomonas* Epi8 into Ad5 HVR5 (AdZ.Epi8) [3]. BALB/c mice immunized with the capsid-modified vectors showed an increase in antibody response consisting of both anti-*pseudomonas* IgG1 and IgG2a subtypes. In addition, mice immunized with the vector containing the OprF epitope were subjected to pulmonary challenge with *pseudomonas*, 60% to 80% of them survived. Zhong and colleagues have utilized the antigen display strategy to incorporate antigens within human adenovirus type 3 hexon [32]. Ad3-based vectors that can present epitopes/antigens to the immune system can be used for multivalent vaccine design. One similar study compared to our work herein is performed by Shiratsuchi et. al, 2010. In this study they were able to incorporate a B-cell malaria antigen within hexon HVR1 in combination with the HI loop of the fiber domain. Although, this approach does not allow equal antigen response due to the differing amounts of fiber and hexon molecules found per virion it could allow for very significant vector manipulation, as seen in similar studies [16], [33]. Most importantly, Shiratsuchi and group found that of all the sites tested in their study (HVR1, HVR5, fiber, and HVR1 and fiber in combination), HVR1 was the best place for malaria B-cell epitope insertion to induce (QGPGAP)_3_ epitope-specific antibody but also protective antibody against the malaria parasite, which ultimately lead to the protection against malaria.

Multivalent Ad vectors such as these can be used to vaccinate and elicit a varied and broad humoral response against pathogens. With the vast diversity of many bacterial pathogens and viral pathogens, such as HIV, the need remains for vaccine vectors that yield a broad and diverse immune response. Successful HIV vaccination remains a tremendous challenge because HIV-1 vaccine strategies must contend with the enormous global sequence diversity of HIV-1 among many other obstacles. To attempt to overcome the obstacle related to mounting a diverse immune response, mosaic vectors and Ad vectors schemes that utilize “heterologous inserts” in prime-boost regimens have been developed in order to increase the breadth and depth of cellular immune responses in nonhuman primate models [11], [15]. These vectors have shown promise; however, these constructs focused primarily on cellular immunity. It is likely that the most successful prophylactic HIV-1 vaccine will elicit a broad and robust cellular and humoral response. In order to produce vectors that could provide a varied humoral response we generated multivalent proof-of-concept vectors.

Our proof-of-concept vectors provide dual B-cell epitope presentation within the Ad capsid protein, hexon. These vectors can be designed to present antigens as transgenes in a similar fashion as in our 2010 study (Matthews et. al, 2010). Currently, we have not evaluated the B-cell epitopes presented within the Ad capsid (i.e. antigen capsid-incorporation strategy) for their impact on cellular immune response. However, in our 2010 manuscript we did evaluate the antigen capsid-incorporation strategy in combination with transgene expression of the HIV gene, gag. Gag expression was not detrimentally impacted by that of hexon capsid modifications. As previously discussed, within the Ad5 platform we can incorporate up to 53–83 amino acids within hexon HVR2 or HVR5. Based on our preliminary studies (data not shown), we speculate that this will be the range of epitopes that can be incorporated within HVR1. We have demonstrated that we can incorporate up to 3 kb within the minor capsid protein IX. Since the size of an antigenic epitope processed within the immune system is generally thought to be equivalent to 5–15 amino acids, our platform could be very useful. If a multivalent Ad vector is generated with epitopes within the hexon and pIX domains this would represent a minimum of 960 antigenic epitopes within one Ad particle. One limitation to our system is the generation of vector immunity; this is common with some viral vectors. Our multivalent vectors will be evaluated for their abilities to escape Ad5 pre-existing immunity.

Overall, our current study demonstrates innovative features related to dual hexon-modified vectors that can be used for next-generation multivalent vaccine vectors. Our study shows that dual antigens can be displayed within one Ad hexon particle and that there is a preference for a particular hexon configuration with respect to maximum *in vivo* immune response. These findings have the potential to be a launching pad for multivalent vectors which have a varied humoral response against one organism or pathogen or multiple organisms or pathogens.
